# Mr. Benjamin Wainewright

**Published:** 1910-09-10

**Authors:** 


Mr. Benjamin Wainewright, of Park Street, Grosvenor Square, has died suddenly at Pontresina, Switzerland. He was educated at the University of Edinburgh, where he graduated M.B., C.M., in 1880, three years later qualifying as a Fellow of the Royal College of Surgeons of England. He was consulting ophthalmic surgeon to the Medical Aid Society, surgeon to Charing Cross Hospital and the Royal Westminster Ophthalmic Hospital, and surgeon in charge of the aural department at the West London Hospital. Formerly he had been senior resident surgeon at the Edinburgh Royal Infirmary and Demonstrator of Anatomy of the University of Edinburgh. Mr. Wainewright, who was fifty-seven years of age, was a member of the Alpine Club and a great climber.

